# Surveillance of the armed forces as a sentinel system for detecting adverse effects of dietary supplements in the general population

**DOI:** 10.1017/S1368980017003111

**Published:** 2017-11-20

**Authors:** Harris R Lieberman, Krista G Austin, Emily K Farina

**Affiliations:** 1 Military Nutrition Division, US Army Research Institute of Environmental Medicine, General Greene Avenue, Building 42, Natick, MA 01760, USA; 2 Oak Ridge Associated Universities, Oak Ridge, TN, USA

**Keywords:** 1,3-Dimethylamylamine (DMAA), Ephedra, Sympathomimetic, Cardiovascular, Food and Drug Administration

## Abstract

**Objective:**

Half the US population takes dietary supplements, but surveillance systems available to regulatory and public health authorities to determine whether specific dietary supplements present a risk are inadequate and numerous severe injuries and deaths have occurred from their consumption. Uniformed military personnel regularly use dietary supplements and are more likely to use potentially dangerous supplements than civilians. Recently, the supplement 1,3-dimethylamylamine (DMAA) was marketed for physical performance-enhancement and weight loss. However, after over 100 reports of illness attributed to DMAA, including six deaths, the Food and Drug Administration issued a warning to cease its sale.

**Design:**

When DMAA was legal (2010–2011), we conducted, using convenience samples, supplement surveys of service members and determined prevalence of use and self-reported symptoms of DMAA use.

**Subjects:**

We surveyed 4374 armed forces personnel using a standardized dietary supplement survey administered by local health-care professionals.

**Results:**

Overall, 11 % of survey respondents used dietary supplements labelled as containing DMAA at least once/week. Regular users were over two times more likely to report tachycardia (*P*<0·0001), tremors (*P*<0·0001) and dizziness (*P*=0·0004), and over three times more likely to report numbness/tingling (*P*<0·0001) than non-users.

**Conclusions:**

Military services could readily monitor adverse events associated with dietary supplements using electronic surveys and medical records. Since armed forces personnel are much more likely than civilians to use potentially dangerous dietary supplements like DMAA, near real-time surveillance of them using electronic surveys and medical records would provide early warning to regulatory agencies and the medical and public health communities when high-risk dietary supplements are introduced.

Half of the US population regularly takes dietary supplements, but ensuring they are safe has proven difficult and contentious since the Dietary Supplement Health and Education Act of 1994 (DSHEA) became law^(^ ^1^ ^–^ ^3^ ^)^. This law significantly reduced regulatory oversight of dietary supplements. As a consequence, it led to a rapid increase in dietary supplement availability and consumption, in part because manufacturers were not required to demonstrate the safety and efficacy of their products. Currently, over 85 000 dietary supplements are on the market and an estimated $US 41·1 billion of them were sold in a recent year^(^ ^4^ ^,^ ^5^ ^)^.

Surveillance systems available to determine whether dietary supplements present a risk to the population are inadequate and numerous severe injuries and deaths have occurred from dietary supplement consumption^(^ ^6^ ^)^. The Food and Drug Administration (FDA) regularly recalls dietary supplements because of safety concerns and adulteration, including unintentional contamination. It was recently reported that approximately 23 000 emergency room visits each year in the USA are attributable to adverse events associated with dietary supplement use^(^ ^7^ ^)^. Most information the FDA receives on potentially dangerous dietary supplements is obtained as anecdotal information submitted by clinicians, consumers or manufacturers using the FDA’s adverse event reporting system and an occasionally pertinent scientific publication. Only about one in 100 adverse events that occur due to dietary supplement use is reported to the FDA and many reports lack sufficient information to be useful^(^ ^6^ ^)^.

From 2006 to 2011 we conducted anonymous, voluntary surveys of dietary supplement use of over 4000 active duty US Armed Forces personnel including reports of the adverse events they experienced. Over half (53–82 %) of service members reported regularly using dietary supplements and service members were much more likely to use purported performance-enhancing dietary supplements than civilians^(^ ^8^ ^–^ ^11^ ^)^. As we conducted these surveys, a dietary supplement, 1,3-dimethylamylamine (DMAA), was aggressively marketed for physical performance-enhancement and weight loss, purportedly by increasing intensity of workouts and effectiveness of weight training^(^ ^12^ ^)^. This ingredient, DMAA, was marketed as a dietary ingredient based on apparently false claims it occurred naturally in geraniums (spp. *Geraniaceae*). One of the regulatory requirements of DSHEA is that any product sold as a dietary supplement be a naturally occurring substance^(^ ^12^ ^,^ ^13^ ^)^. Surprisingly, nearly 50 years ago, DMAA was marketed as an FDA-approved inhaled nasal decongestant by Eli Lilly and Company under the tradename Forthane™. Like many drugs for this and other indications, it is a sympathomimetic compound similar in structure to phenylephrine, amphetamine and ephedra^(^ ^12^ ^–^ ^14^ ^)^. According to the FDA and several peer-reviewed reports, adverse effects reported as a consequence of DMAA use include cardiac arrest, haemorrhagic stroke and death^(^ ^15^ ^–^ ^17^ ^)^. In one recent case report, cardiac arrest was reported to have occurred after consumption of a product that contained DMAA but was not labelled as such^(^ ^15^ ^)^. Furthermore, consumption of several DMAA-containing supplements has been reported to be associated with severe liver injury, in several instances requiring a liver transplant^(^ ^18^ ^)^.

Several animal studies have been conducted to examine various effects of DMAA^(^ ^19^ ^,^ ^20^ ^)^. In one rodent study, acute administration of a DMAA-containing supplement increased running time and distance^(^ ^19^ ^)^. However, chronic administration (4 weeks) of the supplement impaired exercise performance. The authors concluded the supplement had acute stimulant-like effects on rodents consistent with the pharmacological profile of DMAA and its reported acute effects in man^(^ ^19^ ^)^. In another rodent study, DMAA produced effects on behaviour similar to those produced by cocaine and to a lesser extent methamphetamine^(^ ^20^ ^)^.

Several years ago, ephedra was marketed as a dietary supplement for purported performance-enhancement and weight loss like DMAA, and over three billion doses were consumed a year before it was banned in 2004 after nearly 15 000 adverse events associated with its use, including multiple deaths, were reported^(^ ^12^ ^,^ ^21^ ^)^. It appears DMAA was introduced and marketed as a replacement for ephedra, given ephedra’s widespread popularity. It is not surprising that since they have common mechanisms of action and potency, the safety issues and regulatory history of ephedra repeated itself with DMAA^(^ ^12^ ^,^ ^22^ ^)^. In 2013, the FDA issued warning letters to manufacturers of DMAA-containing products to cease its manufacturing and sale after receiving over 100 reports of illness, including six deaths, in both the general population and military service members^(^ ^22^ ^)^. However, due in part to the failure of existing surveillance systems, it took over 16 months for the FDA to take definitive action on DMAA-containing products. Recently, dietary supplement manufacturers have marketed other products that may be dangerous, appear structurally similar to DMAA and are sympathomimetics, such as 1,3-dimethylbutylamine^(^ ^23^ ^)^. Therefore, the objective of the current cross-sectional study was to determine whether there was an association between use of dietary supplements containing DMAA and self-reported adverse events among armed forces personnel.

## Methods

### Participants and survey administration

From 2010 to 2011, when DMAA was legal and readily available, armed forces personnel at thirty-three installations within the USA and overseas were surveyed with a standardized paper-and-pencil survey that has been described previously^(^ ^8^ ^–^ ^11^ ^,^ ^24^ ^)^. Participants completed the survey after a clear explanation that all information obtained would remain confidential and participation was voluntary. The study was approved by the Human Use Review Committee of the US Army Research Institute of Environmental Medicine. Survey sites were chosen based on personnel and health-care provider availability. Personnel on temporary or transitional status, including those who were absent without leave, incarcerated or moving between duty stations, were excluded. Personnel enrolled in training courses that did not permit use of dietary supplements were also excluded. Unit commanders and class leaders identified opportunities to recruit volunteers, such as unit training classes, and coordinated with local health-care providers to provide a standardized recruitment briefing. The briefing described the purpose and contents of the survey and included instructions for completing multipart questions. The survey was completed by 4604 personnel. After excluding participants (*n* 230) with missing data (for age (*n* 69), gender (*n* 66), total number of dietary supplements used (*n* 42), BMI (*n* 24), education (*n* 23) and aerobic exercise (*n* 6)), 4374 personnel were included in the final analytic sample. The sampled population is very similar to the sample (and survey) described previously^(^ ^24^ ^)^. Demographic and lifestyle information (age, gender, education level, BMI, military branch and aerobic exercise) was obtained by the survey.

### Use of 1,3-dimethylamylamine and adverse events

The survey included forty-three questions assessing the type, frequency and reasons for dietary supplement use. Individual supplements listed on the survey included fifty-five generic supplements such as multivitamins, combination antioxidants, and individual vitamin and minerals, as well as thirty-seven brand-named supplements, which were chosen for inclusion in the survey based on patterns of dietary supplement purchases from the Army Air Force Exchange System and General Nutrition Center stores located near military installations. Participants were also instructed to write in supplements they used that were not listed on the survey. Dietary supplements listed on the survey and any other dietary supplements written in by the participant were categorized as a dietary supplement containing DMAA if DMAA was listed by the manufacturer as a product ingredient. These claims were not verified by analytically determining the supplement contents.

The survey question assessing self-reported adverse events was asked separately from questions assessing use of particular dietary supplements and was stated as follows: ‘Have you experienced any of the following negative side effects while consuming dietary supplements?’ Response options included: ‘abnormal rapid heartbeat’, ‘stomach pain’, ‘dizziness or confusion’, ‘tremors or shaking’, ‘numbness or tingling of arms or legs’, ‘loss of consciousness’, ‘other’ or ‘I did not experience any negative side effects’. Participants were instructed to mark all response options that applied.

### Statistical analysis

The statistical software package SAS version 9.2 was used to perform all analyses. Demographic and lifestyle characteristics, the proportion of volunteers using DMAA one or more times weekly and the proportion reporting adverse events were summarized using descriptive statistics, including percentages, means and sd. To determine the association between DMAA use and adverse events, differences in frequencies were determined with a *χ* ^2^ test and the regression of self-reported adverse event occurrence (Y/N) *v*. DMAA use (Y/N) was performed using logistic regression to determine OR and 95 % CI. The crude model (Model 1) was first adjusted for total dietary supplement use (Model 2), and then the additional variables of age, gender, education level, BMI, military branch and aerobic exercise, as well as total number of dietary supplements used, were included in the third model (Model 3). For logistic regression analyses, OR were considered statistically significant when the 95 % CI did not overlap with the null value of OR=1.

## Results

The mean age of the population sampled was 28·5 (sd 7·4) years. Eighty per cent were males, 75 % had at least some college education and 11 % were deployed to a combat theatre. Individuals surveyed were serving in the Air Force (39 %), Army (37 %) or Coast Guard (24 %). On average, personnel spent 5 h/week (297 (sd 221) min) engaged in aerobic exercise, much more than the general civilian population^(^ ^25^ ^)^. Overall, 11 % of those surveyed used dietary supplements labelled as containing DMAA at least once weekly.

Service members using DMAA reported experiencing significantly more adverse events than non-users in crude and adjusted models (Table 1). In the fully adjusted model (Model 3), which included adjustment for use of other dietary supplements, as well as multiple demographic factors, DMAA users relative to non-users were over two times more likely to report tachycardia (OR=2·37; 95 % CI 1·75, 3·20), dizziness (OR=2·17; 95 % CI 1·42, 3·33) and tremors (OR=2·32; 95 % CI 1·67, 3·22), and over three times more likely to report numbness/tingling (OR=3·36; 95 % CI 2·21, 5·12; Table 1). Both tachycardia and tremors are classic side-effects of sympathomimetics like ephedrine, and other adverse events significantly associated with DMAA-containing supplements, such as dizziness, have also been associated with consumption of sympathomimetics^(^ ^12^ ^,^ ^21^ ^)^. Adjusting for other dietary supplements used by service members somewhat attenuated the magnitude of the relationship between DMAA use and adverse events (Model 2), as might be expected. When demographic variables were also included (Model 3), they had only a relatively small effect on the extent of the relationship between DMAA use and specific side-effects (Table 1).

**Table 1:** Associations between self-reported 1,3-dimethylamylamine (DMAA) use and adverse events among US Armed Forces personnel (*n* 4374), 2010–2011. OR and 95 % CI were derived from logistic regression models; users are defined as those consuming DMAA at least one or more times per week

	Adverse event			Model 1*			Model 2†			Model 3‡		
	*n*	%	se	*P* §	OR	95 % CI	*P*	OR	95 % CI	*P*	OR	95 % CI
Tachycardia (abnormal rapid heartbeat)												
Non-user	218	5·59	0·37	<0·0001	3·56	2·71, 4·69	<0·0001	2·48	1·85, 3·34	<0·0001	2·37	1·75, 3·20
User	82	17·41	1·75									
Stomach pain												
Non-user	168	4·30	0·33	0·0007	2·18	1·53, 3·10	0·0652	1·43	0·98, 2·08	0·0815	1·40	0·96, 2·06
User	42	8·92	1·31									
Dizziness												
Non-user	106	2·72	0·26	<0·0001	3·05	2·07, 4·50	0·0002	2·20	1·45, 3·36	0·0004	2·17	1·42, 3·33
User	37	7·89	1·24									
Tremors												
Non-user	188	4·82	0·34	<0·0001	3·22	2·39, 4·34	<0·0001	2·40	1·74, 3·32	<0·0001	2·32	1·67, 3·22
User	66	14·01	1·60									
Numbness/tingling												
Non-user	73	1·87	0·22	<0·0001	5·95	4·08, 8·69	<0·0001	3·54	2·35, 5·35	<0·0001	3·36	2·21, 5·12
User	48	10·19	1·39									
Loss of consciousness												
Non-user	5	0·13	0·06	–		–	–	–		–	–	
User	0	–	–	–		–	–	–		–	–	

* Model 1=crude, unadjusted model.

† Model 2=adjusted for total number of dietary supplements used.

‡ Model 3=adjusted for total number of dietary supplements used, age, gender, education, aerobic exercise, BMI and military branch.

§ Statistical significance of Wald *χ* ^2^ test.

The nature of the adverse events reported by the service members taking DMAA is consistent with a recent report by authors from the Centers for Disease Control and Prevention and the FDA that documented symptoms observed in emergency departments that were attributed to dietary supplements, particularly those such as weight-loss and energy-increasing products that frequently contain various stimulants^(^ ^7^ ^)^. Adverse effects observed in that study included palpitations, chest pain, tachycardia, headache and dizziness and were frequently present in individuals 20–34 years old and associated with consumption of weight-loss and energy supplements^(^ ^7^ ^)^. It should be noted side-effects experienced by consumers using sympathomimetic or other cardiostimulatory dietary supplements may account in part for their popularity. Perceptible physiological changes may convince users the products are working since most dietary supplements do not produce readily noticeable effects. This may encourage manufacturers to include higher doses of stimulants, as well as multiple stimulants, in their products.

## Discussion

Consumption of dietary supplements labelled as containing the sympathomimetic compound DMAA was significantly associated with multiple self-reported adverse events, including tachycardia, tremors, dizziness and numbness/tingling, after adjusting for the use of other dietary supplements and various demographic factors including participation in exercise. Since over 11 % of the sample surveyed were using DMAA as least once weekly, we conservatively estimate that over 150 000 armed forces personnel were taking DMAA regularly when we conducted the surveys. Others may have been inadvertently taking DMAA due to intentional or unintentional contamination of supplements that were not labelled as containing DMAA. Large numbers of civilians, particularly those engaged in extensive weight training, certain athletic activities or attempting to lose weight, were also taking DMAA at that time, and DMAA was in use in other countries such as the UK and New Zealand^(^ ^12^ ^,^ ^16^ ^,^ ^17^ ^)^. Therefore, the risk of DMAA use to various populations must have been substantial.

Given armed forces personnel are much more likely than civilians to use potentially dangerous dietary supplements like ephedra and DMAA, regular surveillance of them could provide early warning that high-risk dietary supplements have been introduced into the marketplace. There are currently over 1·3 million individuals in the active duty US military^(^ ^26^ ^)^. Military services continuously track and communicate with their personnel, so this existing infrastructure, including electronic medical records, could be used to provide real-time epidemiological surveillance of adverse events associated with use of dietary supplements. Dietary supplement surveys, conducted via the Internet and similar to the one we used to detect adverse events associated with DMAA, could therefore be readily conducted on very large, at-risk military populations and immediately cross-referenced to electronic medical records. If these data, especially emergency room and urgent care visits, were integrated with dietary supplement survey data collected from service members in real time via the Internet and evaluated using state-of-the-art medical informatics, the association of specific adverse events with consumption of specific dietary supplements would be possible. The Department of Defense (DoD) currently maintains various surveillance systems for detecting rapidly emerging medical threats, such as epidemics, but systems for continuously monitoring dietary supplement use do not exist currently^(^ ^27^ ^)^.

The DoD has multiple, redundant electronic systems for notifying its personnel and on-base dietary supplement stores of urgent issues. Therefore, once DoD public health officials determined a particular dietary supplement posed a potential hazard, it could be removed from on-base stores and virtually all service members immediately warned to cease its use. Civilian public health officials could also receive this information immediately. Implementing the suggested surveillance system would benefit armed forces personnel and provide early warning to regulatory agencies and the medical and public health communities that potentially dangerous dietary supplements have entered the marketplace.

These data and their interpretation have limitations. Self-reported data are susceptible to report and recall bias. In addition, since the survey was based on a convenience sample it may not be representative of the entire military population, although we did control for various demographic variables in the multiple regression analyses conducted. Reported adverse events and intake of specific dietary supplements were established independently on separate survey questions, so presence of a specific adverse event was not directly associated with a specific dietary supplement by a participant. This is an advantage, as well as a limitation, since it avoided suggesting to volunteers a particular supplement produced specific side-effects and therefore reduced a potential source of bias. It should also be noted that unmeasured confounders, such as heavy strength training, may not have been adequately controlled for which could produce some of the symptoms reported, although we did adjust for intensity of aerobic exercise in Model 3. Another limitation of the study was our inability to analytically verify the DMAA content of the dietary supplements.

## Conclusion

In conclusion, DMAA was frequently used by US military personnel when it was widely available and its regular use was associated with multiple self-reported adverse events, including those associated with use of similar sympathomimetic compounds. A surveillance system of DoD uniformed personnel could provide early warning that dangerous dietary supplements have been introduced into the marketplace.
